# Supplementary material for: Stepwise internal potential jumps caused by multiple-domain polarization flips in metal/ferroelectric/metal/paraelectric/metal stack
Source: Nat Commun. 2020 Apr 20;11:1895. doi: 10.1038/s41467-020-15753-4 (PMC7170928; doi:10.1038/s41467-020-15753-4)
Supplement: Supplementary file 1 — Supplementary Information [file 41467_2020_15753_MOESM1_ESM.pdf]

Supplementary Information

Stepwise internal potential jumps caused by multiple-domain polarization flips in metal/ferroelectric/metal/paraelectric/metal stack

Xiuyan Li et al.

Supplementary Figures

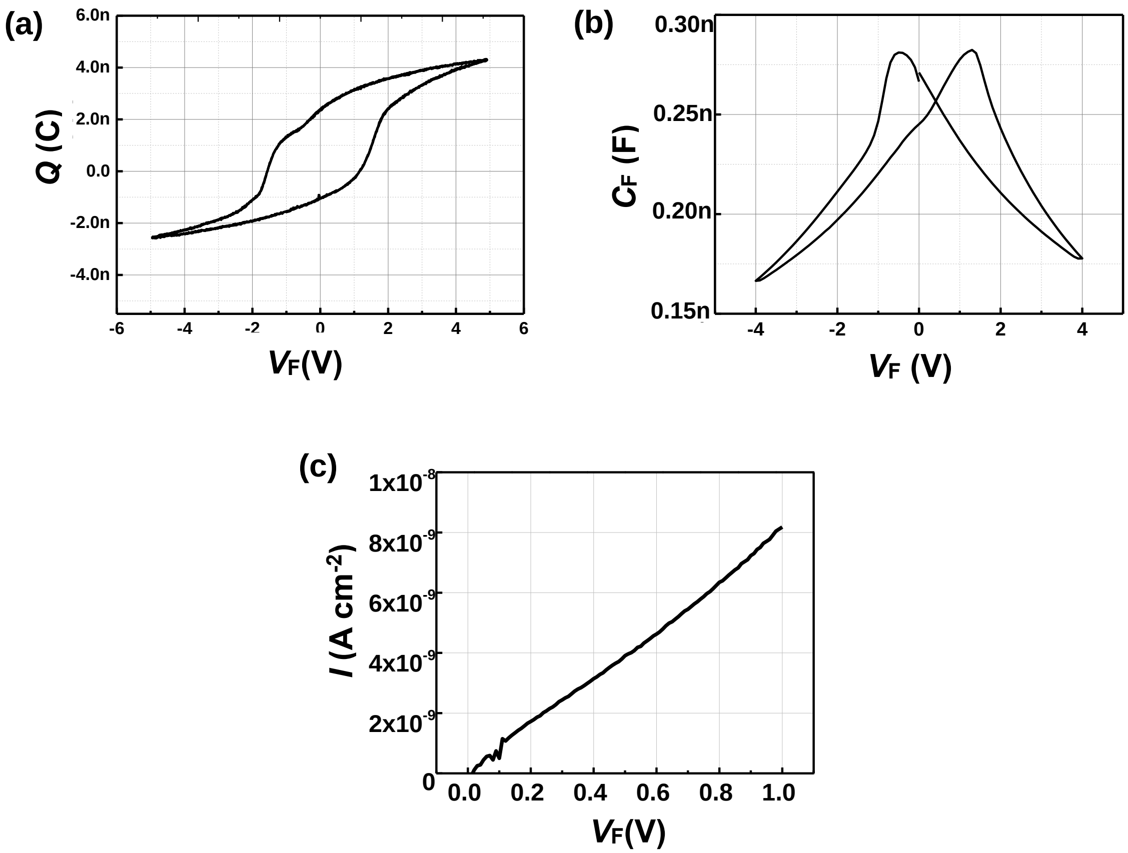

Supplementary Figure 1. Basic characteristics of single PZT-CAP used in this study. (a) Charge-voltage (Q-V_F), **(b)** capacitance-voltage (C_F-V_F) and **(c)** current-voltage (I-V_F) characteristics of a typical PZT capacitor used in this study. Q-V_F characterization was carried at 10 kHz with AC triangular wave with amplitude of V_F. C_F-V_F was measured by superposing an AC voltage of 0.05V in 10 kHz on DC voltage, V_F. I-V_F was measured by sweeping the DC voltage, V_F.

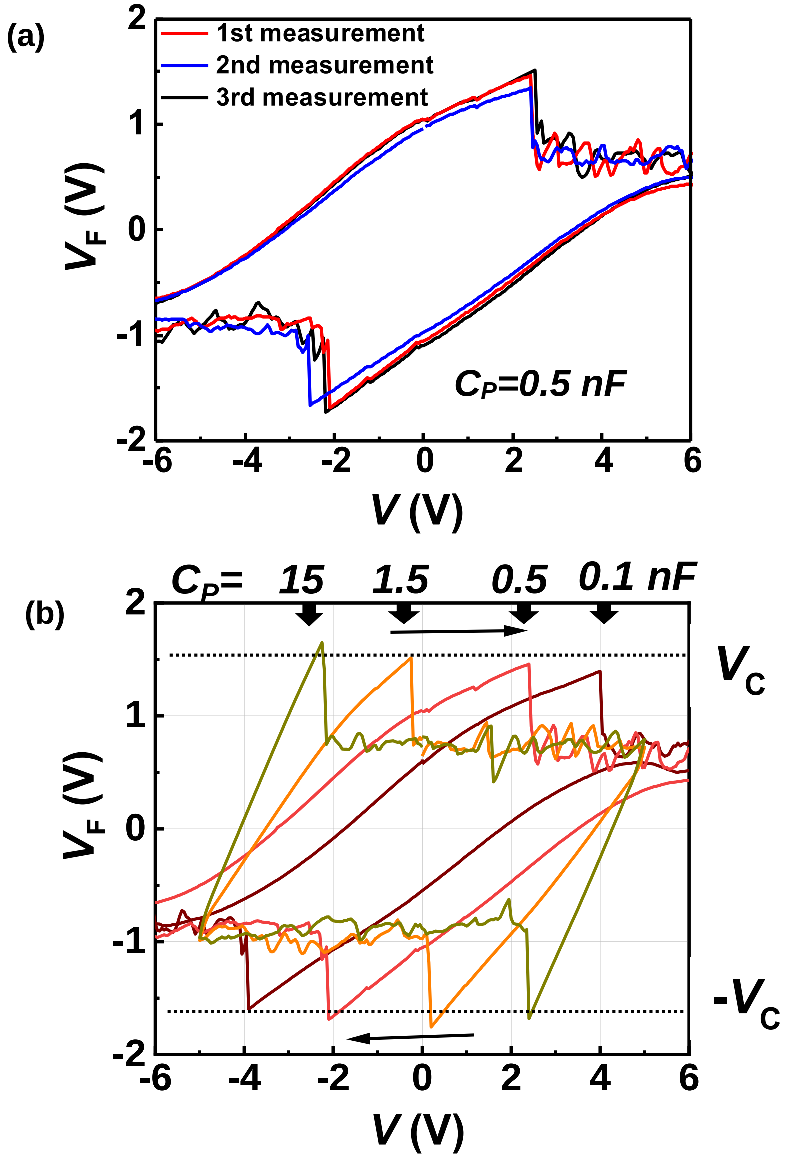

Supplementary Figure 2. Supporting data of V_{int} gain in FE/PE system. V_F-V characteristics **(a)** in FE/PE stack for three consecutive measurements in the same measurement condition and **(b)** in FE/PE stacks with four kinds of C_P. In all cases, the first and biggest V_F drop, corresponding to the first and biggest V_{int} jump, occurs near $\pm V_C$ of FE film. The small zig-zag characteristics following the biggest V_F drop is also reproduced well, though the zig-zag position is not necessarily overlapped among 1st, 2nd and 3rd measurements in (a). This may be due to a possible randomness in the successive domain switching.

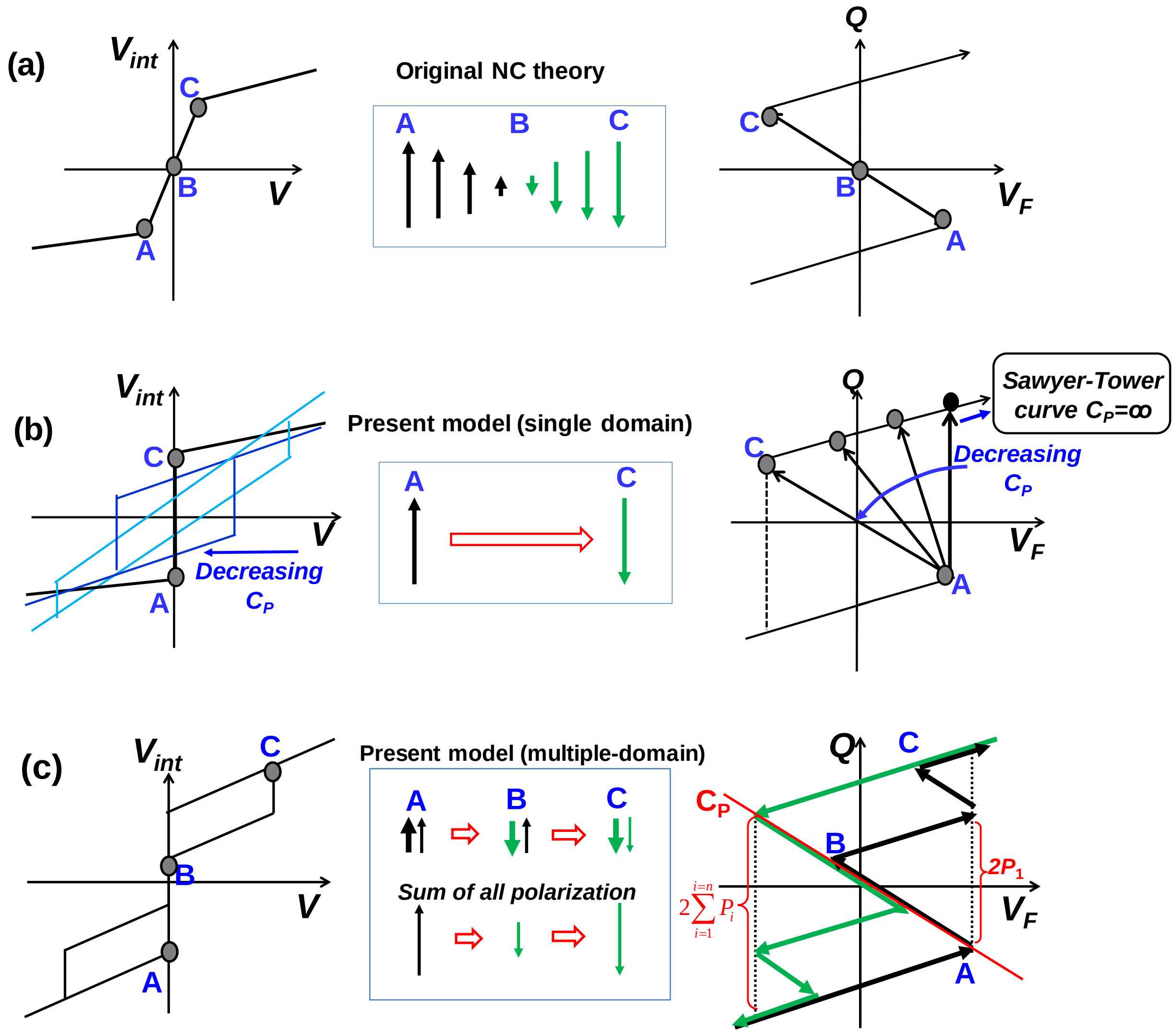

Supplementary Figure 3. Comparison of hysteresis-free V_{int} gain in the present view and in the initial NC proposal. Schematics of possible V_{int} gain in FE/PE stacks, and polarization change and Q-V_F characteristics of FE layer in **(a)** the original NC theory, **(b)** the present view for the case with single domain FE layer and **(c)** the present view for the case with multiple-domain FE layer.

Supplementary Notes

Supplementary Note 1: Internal potential formulation in PE/PE system

The resistance of capacitor is ideally infinite, but it is finite in reality. So, the relevant time response in PE/PE system has been considered for V_{int} formulation. As discussed in the main text, the finite input impedance of measurement system should be also taken into account. Thus, the circuit in Fig. 1(b) in the main text is formulated as,

$$V_1 + V_{\mathrm{int}} = V, \tag{1}$$

$$C_1 \frac{dV_1}{dt} + \frac{V_1}{R_1} = C_2 \frac{dV_{\mathrm{int}}}{dt} + \frac{V_{\mathrm{int}}}{R_2} + \frac{V_{\mathrm{int}}}{R_Z}, \tag{2}$$

where t is the time. In DC mode, V increases in a stepwise manner, V=V_{s} (a constant) at each measurement. Thus, V_{int} is solved as

$$V_{\mathrm{int}} = V_{\mathrm{s}} \left(\frac{C_1}{C_1 + C_2} - \frac{R_2^*}{R_1 + R_2^*} \right) e^{-\frac{t}{\tau}} + \frac{V_{\mathrm{s}} R_2^*}{R_1 + R_2^*}, \tag{3}$$

$$\frac{1}{R_2*} = \frac{1}{R_2} + \frac{1}{R_Z}, \quad \tau = \frac{C_1 + C_2}{\frac{1}{R_1} + \frac{1}{R_2^*}},$$

where τ is a time constant. This suggests that V_{int} based on the capacitance circuit can be only achieved with $t \ll \tau$. In the DC measurement, t is with second scale. In a case with C of ~100 pF, R should be ~10^{11} Ω to achieve $t \ll \tau$. If $t \ll \tau$ is not satisfied, V_{int} should be calculated from accurate impedance analysis by considering the time dependence. On the hand, to measure V_{int} accurately, the effect of R_Z in the measurement system have to be carefully eliminated. In our experiments, resistance of PE-CAP is ~10^{12} Ω. This requires a measurement system with the input impedance >10^{14} Ω.

Supplementary Note 2: Hysteresis of V_{int} gain in FE/PE stacks

Hysteresis-free V_{int} gain and Q-V_F are key characteristics in the original NC effects by assuming FE layer is of single domain (**Supplementary Figure 4(a)**). According to our model of FE/PE system, the V_F drop at V_F=V_C is necessary for achieving the V_{int} gain. In the case with a single-domain FE layer, the V_F drop, δV_F, is quantitatively obtained from Eq. (5) in the main text as

$$\delta V_F = \delta V \frac{C_P}{C_F + C_P} - \frac{2P}{C_F + C_P} . \quad (4)$$

When C_P is extremely large, $\delta V_F \approx \delta V$. This corresponds to the Sawyer-Tower analysis. By decreasing C_P, δV_F<0 is realized with a finite hysteresis. If $\delta V_F = -2V_C$ is achieved by further decreasing C_P, V_F should drop from V_C to -V_C, which suggests that hysteresis-free V_{int} gain and Q-V_F characteristic can be achieved as schematically described in **Supplementary Figure 4(b)**. Since it is reasonably assumed that δV<< $\delta V_F = -2V_C$ in this case,

$$C_P \approx \frac{P - C_F V_C}{V_C} . \quad (5)$$

Although this is the same with the critical condition for stabilizing NC effects in the original theory, a big difference is that the intermediate polarization state between P and -P is not taken into consideration in our model.

The hysteresis control in the case with multiple-domain FE layer is very challenging but practically of a great importance. To find out how to control the subthreshold hysteresis in FET with FE/PE gate stack, the biggest V_{int} gain is focused. Since C_P and P_1 dominantly affect the position and the magnitude of this V_{int} gain, the hysteresis in FET might be minimized by achieving an overlapping region of the biggest V_{int} gain in both forward and backward sweepings if the bias dependence of

semiconductor is ignored, as schematically shown in **Supplementary Figure 4(c)**. This will become possible in the following conditions.

$$C_{\mathrm{P}} \approx \frac{\sum_{i=1}^{i=n} P_i - C_{\mathrm{F}} V_{\mathrm{C}}}{V_{\mathrm{C}}}, \tag{6}$$

$$P_1 > \frac{1}{2} \sum_{i=1}^{i=n} P_i \,. \tag{7}$$

Here, n is the number of domains. The requirement for C_{P} looks similar to that in the single domain case, while that for P_1 is complicated, because the regions showing V_{int} gains in forward and backward sweepings may be overlapped only in the condition of above Eq. (7). Nevertheless, the total sum of polarizations over the successive switching is apparently equivalent to the stabilized intermediate polarization state assumed in the original single domain NC theory. The key difference of the present view from the original NC theory is that the intermediate state of each domain is not stable. As a matter of fact, it is not easy but rather impossible to control the polarization and switching of each domain. In addition, the semiconductor capacitance depends on the electric filed. Thus, hysteresis-free V_{int} gain and Q-V_{F} characteristic are obviously difficult to be achieved in actual FE/PE gate stack FETs.
